# Can erythrocytes release biologically active NO?

**DOI:** 10.1186/s12964-016-0145-y

**Published:** 2016-09-17

**Authors:** Peter M. Benz, Ingrid Fleming

**Affiliations:** 1 Institute for Vascular Signalling, Centre for Molecular Medicine, Johann Wolfgang Goethe University, Frankfurt, Germany; 2 DZHK (German Centre for Cardiovascular Research) partner site Rhine-Main, 60590 Frankfurt am Main, Germany

**Keywords:** Nitric oxide, NO, Red blood cells, Erythrocytes, eNOS, Platelet inhibition, Hypoxic vasodilation, VASP, Soluble guanylyl cyclase, PKG

## Abstract

Under physiological conditions, endothelial cells and the endothelial nitric oxide (NO) synthase (eNOS) are the main source of NO in the cardiovascular system. However, several other cell types have also been implicated in the NO-dependent regulation of cell function, including erythrocytes. NO derived from red blood cells has been proposed to regulate erythrocyte membrane fluidity, inhibit platelet activation and induce vasodilation in hypoxic areas, but these proposals are highly controversial. In the current issue of *Cell Communication and Signaling*, an elegant study by Gambaryan et al., assayed NO production by erythrocytes by monitoring the activation of the platelet intracellular NO receptor, soluble guanylyl cyclase, and its downstream kinase protein kinase G. After systematically testing different combinations of erythrocyte/platelet suspensions, the authors found no evidence for platelet soluble guanylyl cyclase/protein kinase G activation by erythrocytes and conclude that erythrocytes do not release biologically active NO to inhibit platelet activation.

## Commentary

**Fig. 1:**
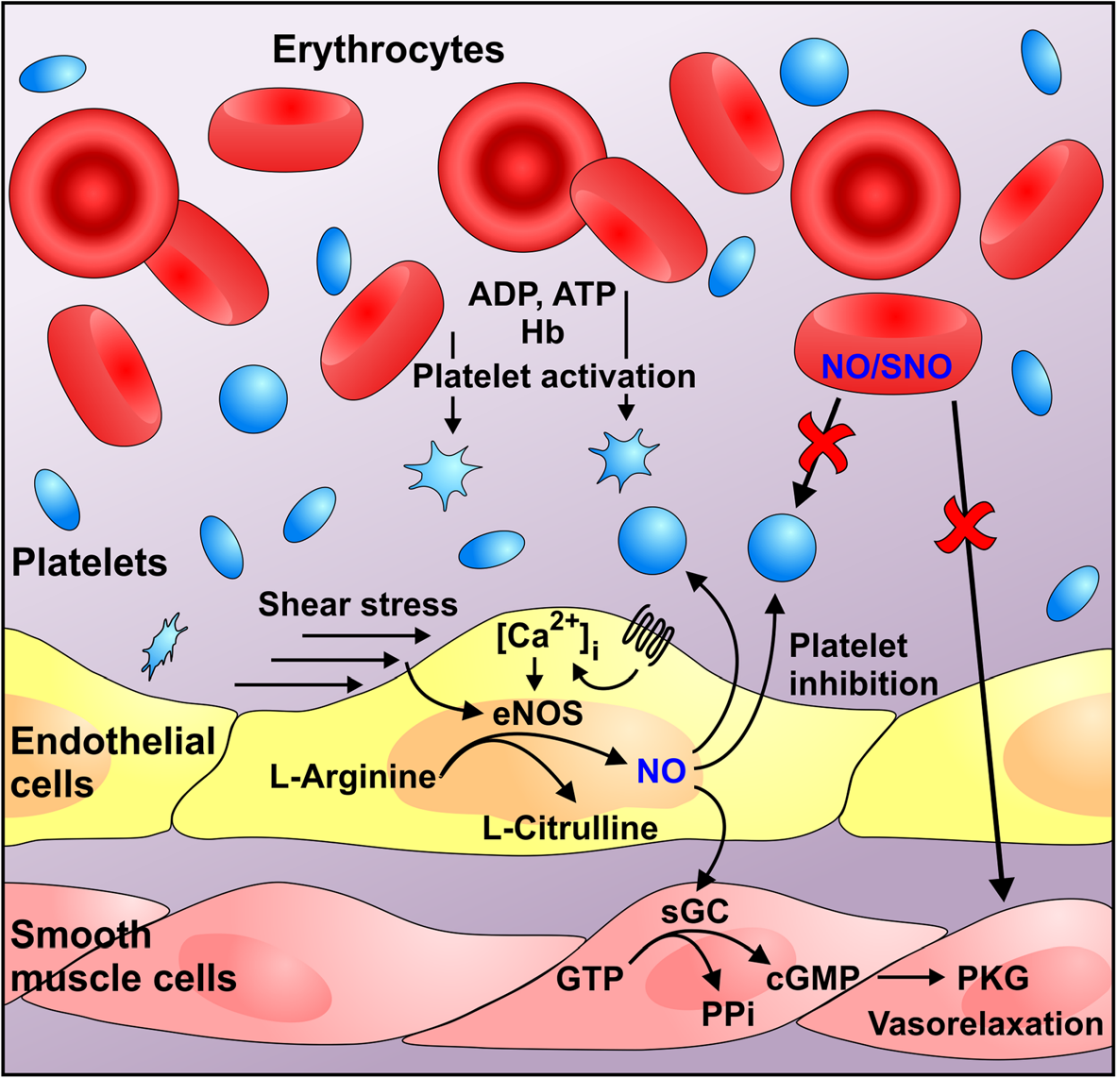
Role of nitic oxide (NO) in blood vessels and functional interplay between erythrocytes and platelets. NO is generated by the endothelial NO synthase (eNOS) upon stimulation by fluid shear stress or Ca^2+^ elevating agonists. The biological actions of NO, including platelet inhibition and smooth muscle relaxation, are mediated by the soluble guanylate cyclase (sGC), which generates cGMP and the subsequent activation of protein kinase G (PKG). Erythrocytes play a role in the regulation of platelet activation as ADP and ATP secreted from damaged erythrocytes directly stimulates platelet purinergic receptors (P2Y_12_, P2Y_1_, and P2X_1_). Secreted hemoglobin (Hb) scavenges endothelial-derived NO and therefore decreases platelet inhibition. It has been proposed that erythrocytes also play a role in platelet inhibition by generation/release of NO or NO-carriers, such as SNO. While it is still debatable whether or not erythrocytes can *generate* NO/SNO, current experimental evidence by Gambaryan et al. concludes that erythrocytes do not *release* biologically active NO/SNO

It is more than 20 years since nitric oxide (NO) was recognized as a biological signalling molecule in the cardiovascular system. NO is generated in endothelial cells by the constitutive endothelial nitric oxide synthase (eNOS), which converts L-arginine into NO and L-citrulline [1, 2]. NO is a short-lived gaseous radical, which diffuses randomly to other cell types, including smooth muscle cells, platelets, and immune cells. Most, if not all, of the biological functions of NO, including platelet inhibition and smooth muscle relaxation, are mediated by the soluble guanylate cyclase (sGC), which converts GTP to cGMP and thereby activates the protein kinase G (PKG) [3–5] (Fig. 1). The activity of eNOS is largely dependent on an increase in the intracellular concentration of calcium [Ca^2+^]_i_, released from the endoplasmic reticulum in response to the activation of receptor-dependent ligands such as acetylcholine, bradykinin, or histamine. eNOS can also be activated, in the absence of a prolonged increase in [Ca^2+^]_i_ by stimuli that elicit the phosphorylation of eNOS on Ser1177. One such stimulus is the fluid shear stress that acts on the luminal surface of vascular endothelium [1, 2] (Fig. 1). In addition to endothelial cells, several other cell types have been reported to generate and release NO; these include vascular smooth muscle cells and cardiac muscle cells. Even though they contain large amounts of the NO scavenger hemoglobin, red blood cells (RBCs, erythrocytes) have also been reported to express eNOS [6] and generate/release NO [7–9] but these observations are still highly controversial.

Erythrocytes and platelets represent the major cell populations in the mammalian blood and clinical observations indicate a functional interaction between the two cell types. On the one hand, there is an undisputed role of erythrocytes in platelet *activation* as bleeding times are prolonged in patients with anemia independent of their platelet count – a condition that can be corrected by erythrocyte transfusion [10, 11]. Importantly, the bleeding defects observed are directly associated with an impaired platelet activation and independent of the blood coagulation system [12]. Furthermore, erythrocyte transfusion can increase platelet activation, which may result in complications in treatment of coronary artery diseases [13]. How erythrocytes contribute to platelet activation is still under debate, but this may involve activation of the ADP-P2Y_12_ receptor pathway and/or the elevated platelet radial movement and interaction with the endothelium [10, 12].

Several recent studies have indicated a role for erythrocytes in platelet *inhibition* by virtue of their ability to release NO [7–9]. Three contradictory mechanisms have been proposed by which erythrocytes may provide NO. The first suggestion was that the nitrosation of a conserved cysteine within the hemoglobin (Hb) β-chain (β93 cysteine) by NO results in the formation of S-nitrosohemoglobin (SNO-Hb), which could function as an NO carrier [14]. The second proposal was that deoxyhemoglobin (deoxy-Hb) may act as a nitrite reductase, and catalyze the formation of NO (and methemoglobin) from inorganic nitrite [15–17]. The third hypothesis is that NO derived from eNOS protein expressed in erythrocytes may directly inhibit platelet aggregation [6]. All of these hypotheses have been the subject of intense debate and all have been challenged by other authors. For example, the generation of a knockin mouse model that replaced hemoglobin β cysteine93 with alanine helped to conclude that SNO-Hb is not essential for the coupling of erythrocyte deoxygenation with increased NO bioactivity in vivo [18]. Moreover, detailed electron paramagnetic resonance (EPR) spectroscopy of nitrite–methemoglobin complexes questioned the proposed role of deoxyhemoglobin in generating NO by the reduction of nitrite [19]. Finally, other authors failed to detect a functional eNOS in erythrocytes [20].

General doubt about the release of NO or NO-carriers from erythrocytes comes from the fact that Hb is an avid scavenger of NO. Notably, physiological NO concentrations range between 100 pM (or below) up to ~ 5 nM, at least six orders of magnitude below the Hb concentration in erythrocytes [21]. Thus while the erythrocyte membrane and the cell free zone established in resistance-sized arteries can reduce the apparent rate at which endothelium-derived NO is consumed by Hb [22, 23], the situation is dramatically different *inside* red blood cells. As outlined above, most of the biological actions of NO, including platelet inhibition and smooth muscle relaxation, are mediated by the sGC/cGMP/PKG pathway (Fig. 1). An important limitation of all of the publications, which claimed platelet inhibition by erythrocyte-derived NO, is that direct activation of the sGC/cGMP/PKG pathway in platelets was not investigated. Together with the conflicting experimental results described above, the ongoing debate about generation and release of NO (or NO carriers) from erythrocytes has precluded a consensus on the physiological role of erythrocytes in platelet inhibition.

In the current issue of Cell Communication and Signaling, Gambaryan et al. [24] set out to solve the discrepancy regarding erythrocyte-mediated platelet inhibition by asking a very simple and logical question. If erythrocytes indeed release physiological relevant levels of NO or SNO to inhibit platelet activation, then the sGC/cGMP/PKG pathway in platelets should be activated, irrespective of the mechanism *by which* NO/SNO are generated in red blood cells. The authors monitored the effects of NO in platelets by assessing the activity of purified sGC in the presence of erythrocytes as well as the NO/sGC/cGMP/PKG-dependent phosphorylation of vasodilator-stimulated phosphoprotein (VASP). The latter is a highly sensitive and reliable assay, used in numerous studies to assess NO-dependent effects and which is widely used in clinical diagnosis of platelet reactivity [25–29]. Other assays that aim to assess the role of NO in platelets, e.g. by the ELISA-base measurement of cGMP, seem to be prone to artefacts, especially in the presence of nitrite or erythrocytes, which both interfere with these assays [30]. Gambaryan et al. systematically tested all possible combinations of erythrocyte/platelet suspensions over time, with varying erythrocyte concentrations, as well as studying erythrocytes containing Hb in different states (oxy-Hb, deoxy-Hb, NO-Hb), with or without nitrite, and platelets isolated under normal or deoxygenated conditions [24]. They found no evidence of platelet and purified sGC activation by erythrocytes. Instead, the authors discovered a strong scavenging effect of NO by erythrocytes - even if NO was added exogenously. Consequently, the authors came to the conclusion that erythrocytes, under all of the conditions tested, cannot inhibit platelet activation by the release of physiological active NO, but in strong contrast act as potent NO scavengers.

At least one additional physiological function has been attributed to erythrocyte-derived NO i.e., the phenomenon of hypoxic vasodilation. According to a hypothesis put forward several years ago [31], erythrocytes have an essential role in matching blood flow to local metabolic demand that can be explained by NO release from Hb at low oxygen saturation of blood Hb to elicit vasodilatation [31, 32]. To-date it is unclear what form this NO has to take to avoid efficient scavenging by oxygen bound to the heme of oxyhemoglobin or with the unoccupied heme of deoxyhemoglobin [33], but NO release form RBCs has been proposed to occur via the reduction of nitrite, the SNO-hemoglobin pathway, as well as endogenous erythrocyte eNOS activity [34]. Even though no contribution to hypoxic vasodilatation was specifically studied, Gambaryan et al. [24] found no evidence for NO/SNO release form RBCs in erythrocyte/platelet suspensions using the sensitive platelet sGC/cGMP/PKG pathway as NO sensor. Given that activation of the same pathway in smooth muscle cells would be mandatory for NO-dependent hypoxic vasodilation, it seems very unlikely that any RBC-derived NO/SNO could diffuse to the much more distant smooth muscle cells.

## Conclusions

In summary, it is still debatable whether or not erythrocytes possess the functional machinery to *generate* NO/SNO. However, irrespective of this question, the study by Gambaryan et al. [24] seems to resolve some of the current controversy by concluding that erythrocytes do not *release* biologically active NO.

## Abbreviations

cGMP: Cyclic guanosine monophosphate
eNOS: Endothelial NO synthase
EPR: Electron paramagnetic resonance
NO: Nitric oxide
PKG: Protein kinase G
RBCs: Red blood cells
sGC: Soluble guanylyl cyclase
SNO-Hb: S-nitrosohemoglobin
VASP: Vasodilator-stimulated phosphoprotein
